# *PLCz* Functional Haplotypes Modulating Promoter Transcriptional Activity Are Associated with Semen Quality Traits in Chinese Holstein Bulls

**DOI:** 10.1371/journal.pone.0058795

**Published:** 2013-03-15

**Authors:** Qing Pan, Zhihua Ju, Jinming Huang, Yan Zhang, Chao Qi, Qin Gao, Lei Zhou, Qiuling Li, Lingling Wang, Jifeng Zhong, Mei Liu, Changfa Wang

**Affiliations:** 1 Dairy Cattle Research Center, Shandong Academy of Agricultural Science, Jinan, PR China; 2 College of Life Science, Nanjing Normal University, Nanjing, PR China; University of Sydney, United States of America

## Abstract

The sperm-specific phospholipase C zeta (*PLCz*) is a candidate sperm-borne oocyte-activating factor that triggers a characteristic series of physiological stimuli via cytoplasmic Ca^2+^ oscillations during fertilization. The molecular mechanisms involved in the regulation of *PLCz* gene expression remain largely unknown. To explore the genetic variations in the 5′-flanking region of the *PLCz* gene and their common haplotypes in Chinese Holstein bulls, as well as to determine whether these variations affect bovine semen quality traits and transcriptional activity, DNA samples were collected from Chinese Holstein bulls and sequenced for the identification of genetic variants in the 5′-flanking region of *PLCz*. Two genetic variants were identified, and their haplotypic profiles were constructed. The two novel genetic variations (g. −456 G>A and g. +65 T>C) were genotyped in 424 normal Chinese Holstein bulls. Bioinformatics analysis revealed that both loci are in transcription factor binding sites of the core promoter region. The association studies revealed that the two genetic variations and their haplotype combinations significantly affected semen quality traits. Using serially truncated constructs of the bovine *PLCz* promoters and the luciferase reporter, we found that a 726 bp (−641 nt to +112 nt) fragment constitutes the core promoter region. Furthermore, four haplotypes, H1H1 (GTGT), H2H2 (GCGC), H3H3 (ATAT), and H4H4 (ACAC), were significantly associated with semen quality traits and successfully transfected into MLTC-1 cell lines. The luciferase reporter assay showed that the different haplotypes exhibited distinct promoter activities. Maximal promoter activity was demonstrated by the H2H2 haplotypes, as compared with the other haplotypes. To the best of our knowledge, this study is the first report on genetic variants and their respective haplotypes in the 5′-flanking region of *PLCz* gene that can influence the semen quality of Chinese Holstein bulls as well as contribute to the transcriptional activity of the *PLCz* promoter.

## Introduction

Fertility is one of the most economically important production traits in cattle improvement. However, fertility is greatly affected by the genetic make-up and the environment. Artificial insemination (AI) is known as one of the most valuable techniques for the genetic improvement of semen quality traits in dairy cattle herds [1]. However, the quantity and quality of semen from bull (including the ejaculate volume, sperm density, initial sperm motility, frozen semen motility, and the sperm deformity rate) are affected by heredity, breeding conditions, the physiological status of the parents, temperature, and other factors. Direct selection for semen quality traits is difficult because of their low heritability [2]. With the progress of genetic and molecular techniques, genetic variations at the DNA level are now key elements for the selection of animals via marker-assisted selection (MAS). Several reports have described the use of candidate genes for MAS to influence semen quality and fertility in boars [3]–[5], goats [6], and bulls [7]–[12].

The sperm-specific phospholipase C zeta (*PLCz*) is a candidate sperm-borne oocyte-activating factor (SOAF) that triggers a characteristic series of physiological stimuli via cytoplasmic Ca^2+^ oscillations during fertilization [13]–[15]. *PLCz* was first cloned from mouse testis [16] and had been sequenced in the human, monkey [17], chicken [18], pig [19], rat [20], and hamster [21] genomes. The initial increase and maintenance of these Ca^2+^ oscillations was observed in the phosphoinositide signaling pathway mediated by the cleavage of phosphatidylinositol 4,5-bisphosphate (PIP2) and the production of 1,4,5-inositol trisphosphate (IP3) [22]–[25]. These data indicate that *PLCz* is an essential protein in the Ca^2+^activity during spermatogenesis and egg activation, which functions as the stimulus for the initiation of normal embryonic development [26], [27].

Daghigh-Kia [28] reported a polymorphism specific to bulls at position 2749 (G>A) in intron 6 of the *PLCz* gene. Their association study between the genotypes and sperm quality traits showed that no significant genotype effects (*P* > 0.05) could be observed in black and red Holstein populations [28]. However, the genetic variants in the 5′-flanking region of the *PLCz* gene have not yet been functionally characterized. Furthermore, little is known about the regulation of *PLCz* expression in bulls. The present study aimed to investigate potentially functional genetic variants in the 5′-flanking region of the *PLCz* gene, to characterize the distribution in a population, and to analyze their effects on semen quality traits in Chinese Holstein bulls. The results indicated that novel molecular markers associated with semen quality traits can be used for MAS in bull breeding programs.

## Materials and Methods

### Animals and cell lines

Semen samples were collected from a total of 424 Chinese Holstein bulls from three bull stations, namely, 142 bulls from the Beijing Dairy Center, 218 bulls from the Shanghai Bright Dairy and Food Co., Ltd., and 64 bulls from the Shandong OX Bio-Technology Co., Ltd. Semen sample collection was permitted by the owners of the animals. Samples were collected by employees of the respective companies. This study was approved by the Bureau of Animal Husbandry and Veterinary and the Dairy Cattle Frozen Semen Quality Supervision Testing Center of the Chinese Ministry of Agriculture.

The 424 genotyped bulls were sons of 230 sires (each sire had 1 to 10 sons, average of 1.84 sons). Repeated measurements of the sperm quality traits from 2007 to 2011 were available for each bull. Data on 40121 ejaculates from the 424 bulls were used, with 4 to 379 ejaculates from each bull. Semen was collected from each bull at 3 d to 6 d intervals using an artificial vagina. Immediately after collection, the ejaculates were stored at 37°C in a water bath prior to evaluation of the fresh semen quality traits. These traits included the semen volume per ejaculate (in ml), sperm motility (in%), sperm concentration (expressed as ×10^8^/ml), and the percentage of abnormal sperm (in%). The fresh semen was then diluted with a glycerol–egg yolk–citrate mixture in the Beijing and Shanghai bull stations or with Bioxcell (IMV Biotechnology, L'Aigle, France) in the Shandong bull station. The collected semen were packaged in 0.25 ml straws and cryopreserved. After storage in liquid nitrogen for 5 d to 7 d, two straws were randomly obtained from each ejaculate and thawed at 38°C for 20 s. These samples were immediately evaluated for the frozen semen quality traits, including post-thaw cryopreserved sperm motility and the percentage of abnormal sperm. The traits were measured using light microscopy, according to the guidelines of the World Health Organization.

Briefly, the ejaculate volume was obtained by dividing the weight of the ejaculate (the difference in weight of a semen-collecting vial before and after sampling) by the semen density [7]. The motilities of the fresh and post-thaw cryopreserved sperm were viewed on a TV monitor connected to a camera mounted onto a phase-contrast microscope (Olympus-BX40; Optical Co., Ltd.) at 400× magnification. A drop of semen was placed onto a pre-warmed (37°C) slide and overlaid with a cover slip. The sperm concentration was determined using a sperm densitometer (Accucell; IMV Biotechnology, L'Aigle, France) that was calibrated using the hemocytometer method. The percentage of viable sperm was calculated by examining more than 100 sperm cells stained by eosin Y-aniline blue from each sample at 400× magnification [29]. The percentage of sperm deformities were similarly calculated at 400× and 1000× magnification with Giemsa staining. To minimize sampling error, the quality trait assessment of all semen samples was performed by a single well-trained technician in each semen collection station.

The semen quality traits included the ejaculate volume, initial sperm motility, sperm density, frozen semen motility, and sperm deformity rate. The means and standard errors of the sperm quality traits investigated in the 424 Chinese Holstein bulls are shown in Table 1. The murine Leydig tumor cells (MLTC-1) were obtained from the Cell Culture Collection of the Chinese Academy of Sciences, Shanghai, China.

**Table 1:** Mean and standard error (SE) of sperm quality traits in Chinese Holstein bulls.

Traits	Mean±SE
Ejaculate volume (ml)	7.22±0.09
Initial sperm motility (%)	80.32±0.61
Sperm density (×10^8^/ml)	11.79±0.16
Frozen/thawed sperm motility (%)	40.92±0.32
Deformity rate (%)	14.43±0.18

### Genetic variants screening of 5′-flanking region

DNA was extracted from sperm using a high-concentration-salt protocol and subsequently stored at -20 °C prior to use. One primer pair (TA4 F/R, Table 2) was designed using primer PREMIER 5.0 to clone the 5′-flanking region based on the GenBank reference sequence (Accession No. AC_000162.1). The primer pairs were synthesized by Shanghai Sangon Biological Engineering Co., Ltd. The DNA amplification fragments from 50 randomly selected samples were directly sequenced in both directions using an ABI PRISM 3730 DNA analyzer (Applied Biosystems, USA) following standard protocol. The sequencing results were analyzed using the DNASTAR 5.0 package (DNASTAR, Inc., USA) to detect genetic variations in the *PLCz* gene.

**Table 2:** Sequences and positions of primers used for cloning the bovine *PLCz* promoter region.

Name	Position	Sequence*
TA1-F	−614 to −596	5′-GGGGTACCTGGTCGTTCACATACAGC-3′
TA1-R	+197 to +213	5′-CCGCTCGAGCCCAAAGCGTCAAAGA-3′
TA2-F	−1195 to −1176	5′-GGGGTACCAAATACCTCAGTGCCTCCC-3′
TA2-R	+94 to +112	5′-CCGCTCGAGGGCTGTTCTGGAGGCTAA-3′
TA3-F	−1979 to −1963	5′-GGGGTACCGCAGCCTAATGACAAA-3′
TA3-R	+241 to +259	5′-CCGCTCGAGGACAGTTATTCTGACGGT-3′
TA4-F	−2290 to −2272	5′-GGGGTACCCTTTAGAGCAGCGGAGTC-3′
TA4-R	+93 to +111	5′-CCGCTCGAGGCTGTTCTGGAGGCTAAG-3′

* The *Kpn*I sites (GGGGTACC) were added to the primers as recognition and protection sites. Reverse primers (3′) contained the *Xho*I site (CCGCTCGAG).

### Prediction of the core promoter region and the functional elements of the 5′-flanking region

The core promoter of bovine *PLCz* was predicted using the Genomatix software (http://www.genomatix.de/). The position of the TATA box was predicted using the Hamming-Clustering Method for TATA Signal Prediction in eukaryotic genes (http://zeus2.itb.cnr.it/~webgen/wwwHC_tata.html). Transcription factors were predicted using WWW Promoter Scan (http://www-bimas.cit.nih.gov/molbio/proscan/) and TFSEARCH (ver. 1.3) (http://www.cbrc.jp/research/db/TFSEARCH.html). Nucleic acid sequences were analyzed using the accepted software formats.

### Genotyping and association study

Sequencing revealed two novel genetic variations (g. −456 G>A and g. +65 T>C) in the 5′-flanking region of the bovine *PLCz* gene. The gene was subsequently genotyped using the polymerase chain reaction (PCR)-restriction fragment length polymorphism (RFLP). The corresponding restriction endonucleases (*Hph*I and *Bsen*I) were selected to digest PCR products according to the manufacturer's recommendations. The digested fragments were separated by polyacrylamide gel electrophoresis (PAGE) using 10% polyacrylamide gels (80 mm×73 mm×0.75 mm). Electrophoresis was performed in 1× TBE buffer at a constant voltage of 120 V for 4 h at room temperature. Gels were stained with 0.1% silver nitrate, and the genotypes were divided into different haplotype patterns.

The SHEsis software (http://analysis.bio-x.cn) was used to analyze the pairwise linkage disequilibrium and haplotype frequencies [30] of the sample population. The association of the identified genetic variations and haplotype combinations with the semen quality traits was analyzed by the least squares method as applied in the “PROC GLM” procedure of SAS software (SAS Institute Inc., Cary, NC, USA), according to the following linear model:



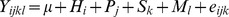



where *Y_ijkl_* is the observed value of each semen quality trait, *µ* is the overall mean, *H_i_* is the fixed effect of genotype or haplotype combinations, *P_j_* is the fixed effect of age (*j* = 2 to 10; classified as: (1) 2 y to 3 y; (2) 4 y to 5 y; (3) 6 y to 10 y); *S_k_* is the fixed effect of the origin of bull (*k* = 1 to 10; classified as: (1) 1 bull from one sire; (2) 2 bulls from one sire; (3) ≥3 bulls from one sire), *M_l_* is the effect of farm, and *e_ijkl_* is the random residual error. Values with *P*<0.05 and *P*<0.01 were regarded as significant. Multiple comparisons were performed using Duncan's test.

### Cloning and construction of *PLCz* reporter plasmid

Bioinformatics analysis showed that the two genetic variations were located within the proximal region of the promoter. To evaluate the promoter activity of different parts of the 5′-flanking region of the *PLCz* gene, we performed serial truncations of the *PLCz* promoter fragment, ranging from −2290 bp to +213 bp, and analyzed the activity of the reporter constructs. The four pairs of primers that were progressively located closer to the transcription starting site of the *PLCz* gene are listed in the Table 2. The forward and reverse primers contained restriction sites for *Kpn*I and *Xho*I, respectively. The *Kpn*I sites (GGGGTACC) were added to primers as recognition and protection sites. The reverse primer (in the 3′ direction) contained an *Xho*I site (CCGCTCGAG). The amplified promoter fragments were purified, double-digested with the restriction enzymes, and cloned into the pGL3-Basic Luciferase Reporter Vector (Promega, Beijing, China). The recombinant plasmids were confirmed by sequencing, were confirmed by sequencing, namely, pGL3-TA1, -TA2, -TA3, and -TA4.

To examine the effect of different haplotypes on *PLCz* promoter activity, a series of reporter plasmids with different haplotypes encompassing the TA4 fragments were constructed. The resulting constructs were named pGL3-H1, pGL3-H2, pGL3-H3, and pGL3-H4. Each plasmid harbored a core promoter region with different haplotypes.

### Transient transfection and luciferase reporter assays

The MLTC-1 cell line was cultured in RPMI-1640 medium (Sigma Co., St. Louis, MO, USA) with 10% fetal bovine serum (Invitrogen Life Technologies) containing 10 mg/L of penicillin and streptomycin (Invitrogen Life Technologies) at 37°C in 5% CO_2_. For the luciferase reporter assays, MLTC-1 cells were inoculated in 48-well plates and grown to 70% to 80% confluence. Transfection was performed using the Lipofectamine^TM^ 2000 reagent (Invitrogen) according to the manufacturer's instructions. Cells were cotransfected with 50 ng of the pRL-TK vector DNA (Promega) and 400 ng of either the empty pGL3-Basic plasmid (a promoterless control from Promega) or one of the promoter constructs with different lengths and haplotypes of the *PLCz* promoter. The pRL-TK vector, which provided the constitutive expression of *Renilla* luciferase, was cotransfected as an internal control to correct for differences in transfection and harvesting efficiency. After 36 h of incubation, cells were harvested and analyzed for luciferase activity using the Dual-Luciferase Reporter Assay System (Promega). Promoter activity was reported in relative light units (RLUs) and normalized against the activity of the empty pGL3-Basic vector. All transfections were performed in triplicate and repeated at least thrice in independent experiments.

## Results

### Identification of genetic variants within the 5′-flanking region of *PLCz* gene

**Figure 1:**
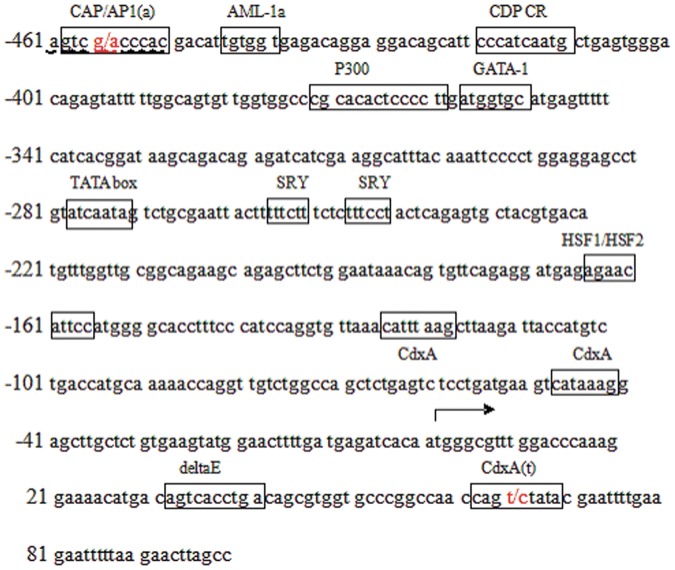
5′-Flanking sequence of bovine *PLCz* from −461 bp to +100 bp. This fragment contains the promoter exon 1 and a part of intron 1. Boxed sequences represent the putative transcription factor binding sites. The nucleotides highlighted in red represent the two polymorphic sites in Chinese Holstein bulls. The nucleotide sequence numbered +1 is A of the TSS. The numbering of nucleotides is relative to the transcription start site.

We sequenced a 1836 bp segment from the 5′-flanking region of the *PLCz* gene in Chinese Holstein bulls. Compared with the *PLCz* gene sequence (GenBank accession no. NC_007303.4), two novel genetic variations (g. −456 G>A and g. +65 T>C) were detected (Fig. 1). These two variations have been submitted to the NCBI database (submitted SNP numbers: g. −456 G>A ss 478894128 and g. +65 T>C ss 478894130).

### Prediction of the promoter region of *PLCz* gene

The bioinformatics analysis predictions indicated that the core promoter region of the *PLCz* gene is located in the −461 bp to +100 bp region. Bovine *PLCz* genes do not have a typical TATA box in the promoter region. However, a putative TATA box with an ATCAATA sequence was present at −289 bp to −283 bp from the transcription start site (TSS; the nucleotide sequence numbered +1 is the first A of the TSS) [31]. The putative promoter contained six functional elements, including the AML-1a, CDP CR, P300, GATA-1, SRY, HSF, delta E, and Cdxa transcription factor binding sites (Fig.1).

The SNP g. −456 G>A was found in the transcription factor binding site of the promoter core region. This SNP was in an activating protein-1 (CAP/AP1) transcription factor binding motif, which was eliminated by the presence of the G allele. In the locus g. +65T>C, the “C” allele caused the disappearance of the CdxA transcription factor binding site (Fig. 1). These results were used to investigate the potential effects of the two genetic variations on the regulation of bovine *PLCz* gene transcription.

### Genotyping the novel genetic variations by PCR-RFLP

**Figure 2:**
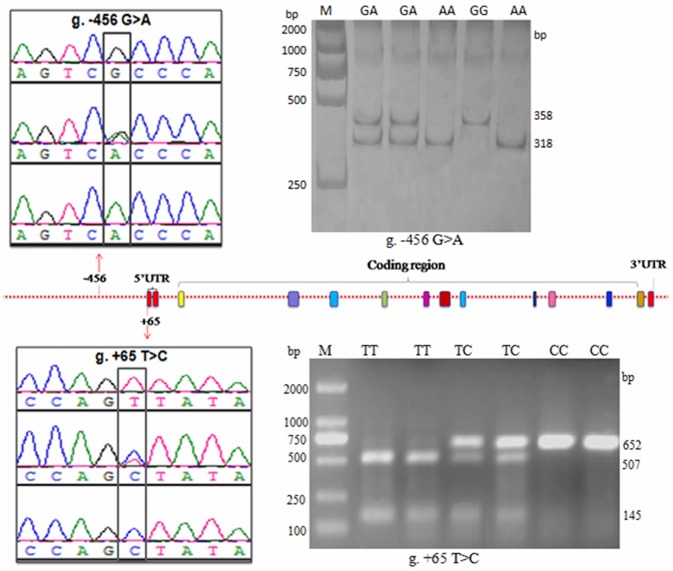
Genetic variation in the 5′-flanking sequence and PCR-RFLP patterns of the two bovine *PLCz* loci. Patterns for g. −456 G>A: genotypes CC, TC, and TT; patterns for g. +65 T>C: genotypes AA, GA, and GG. Digested products smaller than 50 bp are not shown. M: Marker.

Two novel genetic variations g. −456 G>A and g. +65 T>C were genotyped by PCR-RFLP in the 424 Chinese Holstein bulls. Digestion with *Hph*I of the amplified *PLCz* gene g. −456 G>A locus produced fragments of the following sizes: 318 and 40 bp for genotype AA; 358, 40, and 318 bp for genotype GA; and 358 bp for genotype GG (Fig. 2). Digestion with *Bsen*I of the amplified g. +65 T>C locus produced the following fragment sizes: 507 and 145 bp for genotype TT; 652, 507, and 145 bp for genotype TC; and 652 bp for genotype CC (Fig. 2). The obtained genotypes were in agreement with the DNA sequencing results.

### Genetic parameter analysis of two genetic variations

**Figure 3:**
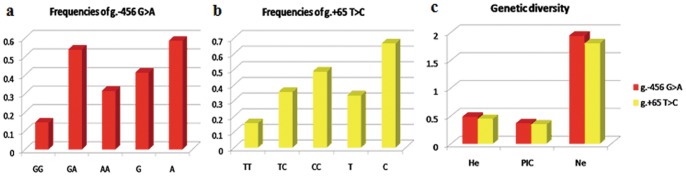
Distribution of genotypic and allelic frequencies of the *PLCz* loci (a) g. −456 G>A and (b) g. +65 T>C in Chinese Holstein bulls. (c) Genetic diversity of *PLCz* in the sample population.

The genotypic and allelic frequencies of the g. −456 G>A and g. +65 T>C loci as well as their genetic diversity are shown in Fig. 3. The A and C alleles are the dominant alleles of g. −456 G>A and g. +65 T>C, respectively. Their high frequencies might be attributed to the long-term breeding of these populations for different purposes and selections. The *PIC*, *H_e_*, and *Ne* values as well as the *χ* ^2^-test results indicated that g. −456 G>A was in Hardy-Weinberg equilibrium (*χ* ^2^_−456_ = 3.29, *P*>0.05) for the analyzed population. Therefore, the selection pressure on this SNP in the population was not powerful. By contrast, g. +65 T>C did not meet the criteria for Hardy-Weinberg equilibrium (*χ* ^2^_+65_ = 12.40, *P*<0.05).

### Association between single variation and semen quality traits in Chinese Holstein bulls

The effects of the two novel genetic variations on semen quality traits (ejaculate volume, sperm density, initial sperm motility, frozen semen motility, and the deformity rate) of Chinese Holstein bulls are summarized in Table 3. g. −456 G>A was associated with the ejaculate volume (*P*<0.01). This locus is situated within a putative binding site for the transcription factor CAP/AP1. Bulls with the genotype GG had higher ejaculate volume than those with genotype AA. Similarly, g. +65 T>C was significantly associated with the ejaculate volume in the analyzed population (*P*<0.05). The presence of the T allele at g. +65 T>C created a CdxA transcription factor binding motif that was eliminated upon its substitution by the C allele. Bulls with the genotype CC had higher ejaculate volume than those with genotypes CT and TT (*P*<0.05). The g. −456 G>A and g. +65 T>C loci are within transcription factor binding sites, which implied their correlation with the transcriptional regulation of bovine *PLCz* via the binding of transcription factors.

**Table 3:** Least square means (LSM) and standard error (SE) for semen quality traits of different genotypes in bovine *PLCz* gene of Chinese Holstein bulls.

Locus	Genotype/Sample	Allele frequency	Ejaculate volume (ml)*	Initial sperm motility (%)	Sperm density (×10^8^/ml)	Frozen/thawedSperm motility(%)	Deformity rate(%)
g. −456 G>A	GG/63	G (0.41) A (0.59)	7.57±0.24^A^	80.72±1.48	11.69±0.46	41.25±0.73	14.47±0.48
	GA/225		7.10±0.13^A^	78.55±0.81	11.75±0.25	40.34±0.40	14.42±0.25
	AA/136		6.68±0.17^B^	78.63±1.05	11.25±0.32	41.37±0.53	14.29±0.37
g. +65 T>C	TT/64	T (0.33) C (0.67)	6.93±0.15^b^	78.92±0.93	11.53±0.28	40.34±0.46	14.58±0.30
	TC/152		6.86±0.17^b^	78.47±1.00	11.26±0.31	41.17±0.50	14.64±0.35
	CC/208		7.48±0.19^a^	79.52±1.16	12.16±0.36	41.03±0.58	13.89±0.35

* Means in the same column with different lowercase superscripts (a and b) are different at *P*<0.05; means in the same column with different uppercase superscripts (A and B) are different at *P*<0.01.

### Association of *PLCz* haplotype combinations with semen quality traits in Chinese Holstein bulls

Four haplotypes were constructed, namely, H1 (GT), H2 (GC), H3 (AT), and H4 (AC). The estimated haplotype frequencies of H1, H2, H3, and H4 were 32.7%, 17.9%, 19.3%, and 30.1%, respectively. Consequently, only eight combinations were used for the population analysis, namely, H1H1 (GTGT), H1H2 (GTGC), H1H4 (GTAC), H2H2 (GCGC), H2H4 (GCAC), H4H4 (ACAC), H1H3 (GTAT), and H3H4 (ATAC).

A number of significant associations were observed between the various haplotype combinations and bovine semen quality traits (Table 4), if the environment and peculiarities in the AI stations were disregarded. The ejaculate volume from bulls with the haplotype combinations H1H1 (*P*<0.05), H1H2 (*P*<0.01), H1H4 (*P*<0.01), H2H2 (*P*<0.05), H2H4 (*P*<0.01), and H4H4 (*P*<0.05) were significantly higher than those with the haplotype combination H3H4. Bulls with the haplotype combination H4H4 demonstrated higher initial sperm motility by 6.15% and 7.23% than the animals with the haplotype combinations H1H3 (*P*<0.05) and H3H4 (*P*<0.05), respectively, but had lower motility than those with H2H2 (*P*<0.05). Bulls with the haplotype combinations H1H2 (*P*<0.05) and H2H2 (*P*<0.05) had significantly higher sperm density than the haplotype combination H3H4. Significant associations were not found among the different haplotype combinations for post-thaw cryopreserved sperm motility and the sperm deformity rate.

**Table 4:** Least square mean (LSM) and standard error (SE) for semen quality traits of different *PLCz* haplotype combinations in Chinese Holstein bulls.

Genotypes combination/Samples*	Ejaculate volume (ml)**	Initial sperm Motility (%)**	Sperm density (×10^8^/ml)**	Frozen/thawed sperm motility (%)	Deformity rate (%)
H1H1/49	7.35±0.30^a^	81.52±1.86	11.33±0.57	41.31±0.94	14.51±0.63
H1H2/47	8.14±0.59^A^	84.51±3.58	13.35±1.11^a^	40.78±1.80	15.45±0.99
H1H4/51	7.56±0.34^A^	80.33±2.06	11.96±0.64	40.80±1.04	14.72±0.73
H2H2/33	7.75±0.52^a^	84.46±3.16^a^	12.68±0.65^a^	41.48±1.59	13.17±1.08
H2H4/39	7.50±0.25^A^	78.57±1.52	11.94±0.47	40.72±0.76	14.02±0.44
H4H4/41	7.31±0.35^a^	83.20±2.12^b^	11.92±0.97	41.47±1.09	13.85±0.70
H1H3/81	6.79±0.17	78.08±1.05^a^	11.60±0.32	40.04±0.53	14.61±0.34
H3H4/83	6.47±0.19^bB^	77.18±1.19^a^	10.80±0.37^b^	41.33±0.60	14.46±0.43

* H1 = GT; H2 = GC; H3 = AT; H4 = AC.

** Means in the same column with different lowercase superscripts (a and b) are different at *P*<0.05; means in the same column with different uppercase superscripts (A and B) are different at *P*<0.01.

### Promoter activity of the *PLCz* 5′-flanking region

**Figure 4:**
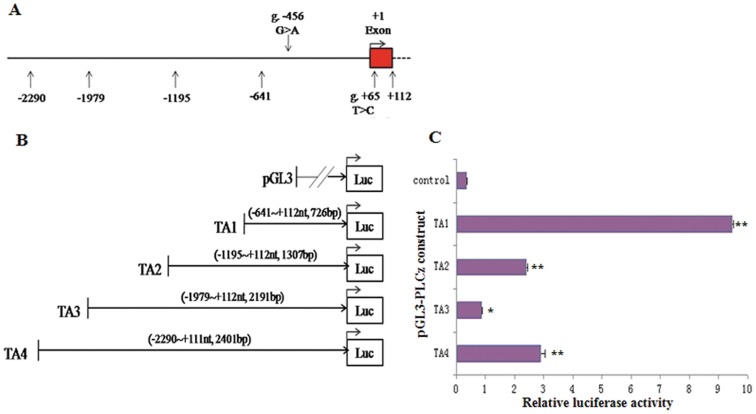
Scheme of the 5′-flanking region of *PLCz* gene and identification of the core promoter region. **A.** 5′-flanking region of bovine *PLCz*, as identified in the NCBI database. The base numbers are relative to the A of TSS. The red box represents exon 1. g.−456 G>A and g.+65 T>C are located in the 5′-flanking region (−2290 nt to +111 nt) of *PLCz* gene. Other numbers represent primer positions for cloning reporter constructs. **B.** Fragments TA1, TA2, TA3, and TA4 were amplified by PCR to produce the reporter constructs; their positions and lengths are shown in parentheses. Each fragment with wild-type alleles was cloned into the pGL3-Basic vector. **C.** Relative luciferase activity of a series of truncated constructs in the *PLCz* 5′-flanking region, as measured by dual luciferase assays of MLTC-1 cells. For each construct, plasmid DNA extracted from 6 to 9 colonies was used. Results are presented as the average fold of firefly luciferase activity versus the *Renilla* control vector (mean±S.D., *n* = 6 to 9). * indicates *P*<0.05, ** indicates *P*<0.01 vs. the pGL3 basic control (Ctrl).

To determine whether the fragment −2290 nt to +111 nt constitutes an active promoter, we amplified a 2401 bp fragment containing the 5′-flanking region of *PLCz* (Fig. 4A). Subsequently, we generated truncated constructs (TA1, TA2, TA3, and TA4) by progressive deletion of nucleotides from the 5′-end and cloned these fragments into the pGL3-Basic Luciferase vector (Fig. 4B). These constructs were transiently transfected into MLTC-1 cells and tested for luciferase activity to determine the shortest required sequence for the transcription of *PLCz*.

As shown in Fig. 4C, the promoter activity of all the constructs was significantly higher than the basal activity of the control pGL3 vector. Sequential additions caused the 5′-end of the 726 bp TA1 fragment to generate different constructs, from TA1 to TA2 (1307 bp), TA3 (2191 bp), and TA4 (2401 bp), thereby decreasing the luciferase activity by approximately 74.7%, 90.8%, and 69.3%, respectively (relative to the shortest construct, TA1). This result indicated that the region between −641 nt and +112 nt is responsible for most of the promoter activity in the *PLCz* gene. Therefore, the TA1 fragment of the proximal 5′-flanking region of *PLCz* was recognized as the active core promoter. The TA1 fragment contained both g. −456 G>A and g. +65 T>C. To investigate the effect of these potential functional genetic variations on the *PLCz* expression, the 5-flanking region of the *PLCz* gene containing these loci was subjected to further functional analysis of the promoter activity.

### Differential transcriptional activity of different *PLCz* promoter haplotypes

**Figure 5:**
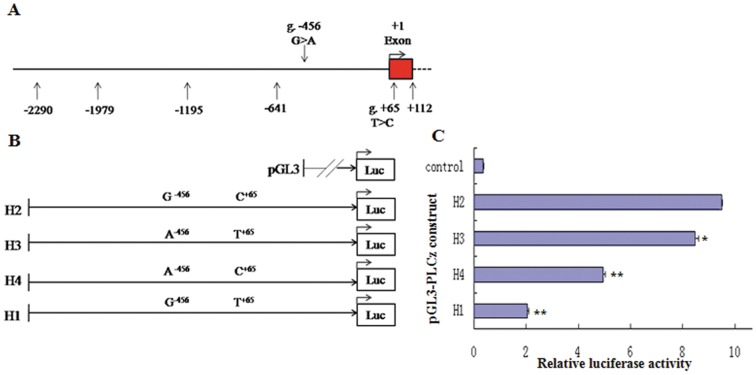
Transcriptional activity of *PLCz* promoter with recombinant haplotypes in MLTC-1 cells. **A.** 5′-flanking region of bovine *PLCz* gene, as identified using the NCBI database. The base numbers are relative to the A of TSS. The red box color represents exon 1. g.−456 G>A and g. +65 T>C are located in the 5′-flanking region (−2290 nt to +112 nt) of *PLCz*. Other numbers represent primer positions for the cloning reporter constructs. **B.** TA1 fragments with different haplotypes (H2, H3, H4, and H1) were amplified by PCR to generate the reporter constructs; the various recombinant haplotypes are shown above the line. Each fragment was cloned into the pGL3 basic vector and transfected into MLTC-1 cells. **C.** Transcriptional activities of the *PLCz* promoter with various haplotypes were measured by dual luciferase assays. For each construct, individual plasmid DNA extracted from 6 to 9 colonies was used. Results are presented as the average fold change of firefly luciferase activity versus the *Renilla* control vector (mean±S.D., *n* = 6 to 9) (* indicates *P*<0.05, ** indicates *P*<0.01 vs. H2).

To assess the effect of the potentially functional variants (Fig. 5A) on the transcriptional activity of the different *PLCz* promoter haplotypes, we generated four haplotype constructs (Fig. 5B), which were designated as H1 (GT), H2 (GC), H3 (AT), and H4 (AC). Different recombined haplotypes from the *PLCz* core promoter TA1 were obtained for transfection into MLTC-1 cells. The dual-luciferase activity of the pGL3-Basic vector alone was used as the control. As shown in Fig. 4C, all the constructs provided higher levels of luciferase expression than the basal activity (control) of the empty vector (pGL3) (*P*<0.05). However, the recombined haplotype constructs differed from each other in terms of their transcriptional activity. Maximal activity was observed for H2 (GC) haplotypes as compared with the others haplotypes. The H2 haplotypes containing the −456 G and +65 C variants showed 80% and 53% higher transcriptional activity, as compared with the haplotypes H1 (GT; containing the −456 G and +65 T variants) and H4 (AC; containing the −456 A and +65 C variants), respectively (*P*<0.01; Fig. 5C). Given that the region between −641 nt and +112 nt represents the core promoter, g. −456 G>A and g. +65T>C may be functional variants involved in the transcriptional regulation of *PLCz*. Taken together, our data indicated that the functional g. −456 G>A and g. +65 T>C variants in the recombined haplotypes played significant roles in the transcriptional activity of the *PLCz* promoter.

## Discussion

The heritability values of the ejaculation volume, sperm concentration, and sperm motility were 0.09, 0.16, and 0.22, respectively [32]. Correlations between breeding values of semen quality traits and routinely estimated breeding values of male fertility were low and ranged from 0.08 to 0.17 [33]. Therefore, single locus effects are expected to be minimal and would require the analysis of larger animal populations [34]. In this study, the g. −456 G>A SNP in *PLCz* was in Hardy-Weinberg equilibrium in the population of 424 bulls, thereby implying that the tested population had constant allele and genotype frequencies.

*PLCz,* which is expressed in mammalian sperm, uniquely possesses all the essential properties of a sperm factor [16]. The bovine *PLCz* gene is composed of 14 exons and encodes a protein with 634 amino acids; PLCz is a key protein during sperm-egg fusion in mammals [35]. The 13 known mammalian PLCs are classified according to their structure into six types (Beta, Gamma, Delta, Epsilon, Zeta, and Eta), of which *PLCz* is the smallest [36]. *PLCz* contains the X and Y catalytic domains, which are associated with the catalytic activity common to all phosphoinositide-specific PLCs, two pairs of EF hand domains that are associated with Ca^2+^ binding, the C2 domain that binds to phosphoinositde containing lipids [36], and a putative nuclear localization signal in the linker region between the X and Y domains. Polymorphisms in the 5′ UTR of a gene may affect its gene product(s) by altering transcription factor binding or RNA stability. To date, little work has been done to investigate potentially functional genetic variants in the 5′-flanking region of the *PLCz* gene and to characterize their distribution in Chinese Holstein bulls. To obtain more information on the regulatory polymorphisms in the 5′-flanking region of the *PLCz* gene, we conducted a functional analysis of the 5′-flanking region of *PLCz*. In the present study, 5′-flanking sequences of the bovine *PLCz* gene were cloned and sequenced to determine the genetic variation in the population and to predict the core promoter and regulatory elements. Furthermore, the novel genetic variations g. −456 G>A and g. +65 T>C were identified in the putative promoter region of the bovine *PLCz* gene.

In bulls, Daghigh-Kia [28] reported a polymorphism at +2749 (G >A) in intron 6 of the *PLCz* gene. DNA variations in introns and exons of *PLCz* were previously reported to have an important effect on male infertility [37]. In the present study, two SNPs were found by screening transcription factor binding sites in the 5′-flanking region of the *PLCz* locus. Genetic variations in transcription factor binding sites may cause significant potential phenotype diversity [38]. Numerous studies have reported that promoter region polymorphisms are associated with gene expression. For example, a promoter haplotype of the mannan-binding lectin (*MBL*) gene was associated with the dramatic decrease in the serum concentration of the gene product [39]. Similarly, a genetic variation (g. −534 T>C) in the bovine peptidase S (*PEPS*) gene promoter was associated with fat percentage and SCS (somatic cell score) traits [40]. In this study, the g. −456 G>A locus was in a transcription factor (CAP/AP1) binding site, which was present with the G allele but lost by the presence of the A allele. CAP/AP1, as a crucial transcription factor, consists of homodimers and heterodimers of jun, fos, and other activating transcription factor proteins. Studies suggest that different AP-1 factors may regulate different target genes and consequently execute distinct biological functions [41]. Therefore, the g. −456 G>A locus can be considered as a regulator of gene expression via transcription factors.

The g. −456 G>A and g. +65 T>C loci with genotypes GG and CC, respectively, were significantly associated with bovine ejaculate volume traits. The haplotype combination H2H2 (H2 = GC) likewise presented relatively high ejaculate volume. These findings have possible interpretations. The ejaculate is composed of secretions from various sources. Approximately two-thirds of the ejaculate volume is contributed by the seminal vesicles, whereas less than one-third is contributed by the prostate. Furthermore, up to 10% could have originated from the testicle and epididymis, with a small fraction from the bulbourethral glands [42]. Spermatogenesis is a complex process involving spermatogonial stem cells. As a surface protein, the equine *PLCz* expression is localized in the acrosome, equatorial segment, and head-midpiece junction, as well as the principal piece of the flagellum, in all epididymal, uncapacitated, and capacitated sperms by immunofluorescence [43]. Thus, *PLCz* might have important functions during spermatogenesis and sperm cell development in the seminiferous tubules and epididymis. Furthermore, the effect of the SNPs g. −456 G>A and g. +65 T>C, specifically the haplotype combination H2H2, on the ejaculate volume traits requires further elucidation. With the application of molecular genetics, the *PLCz* gene associated with semen quality traits can be utilized in breeding programs through MAS. Therefore, the H2H2 haplotype may be used as a molecular marker for the selection of bulls with high ejaculate volume.

This study is the first report that analyzes the transcriptional activity of the 5′-flanking regions of the bovine *PLCz* gene in MLTC-1. MLTC-1 was chosen as the cell line for our experiments because the predicted amino acid sequence of bovine *PLCz* had 69% homology with that of murine *PLCz*, as revealed by the multiple alignment using the ClustalW algorithm. Furthermore, our results support the notion that each fragment has very high transfection efficiency in MLTC-1 cells. The fragment TA1 had a significantly higher effect on the decreasing promoter activity, which is consistent with predicted results of the bioinformatics analysis. However, the TA2 and TA4 fragments had a significantly higher relative luciferase activity, as compared with the pGL3-basis vector. The mechanism for the masking effect of these fragments remains unknown. However, we hypothesize that the complex upstream regulatory elements may interact with each other during the regulation of *PLCz* gene expression. The identification of other signals or extracellular stimuli that interact with the factors that induce changes in the *PLCz* promoter activity would be of interest.

Haplotypes, unlike single genetic variations, are more likely to have significant effects on traits [44], [45]. In this study, the genetic variations g. −456 G>A and g. +65 T>C are located at the core promoter region of the *PLCz* gene, thereby suggesting their important function in bovine *PLCz* expression. The significant associations of the eight haplotype combinations and the semen quality traits varied among the tested Holstein bulls. Furthermore, the haplotypes H1 (GT), H2 (GC), H3 (AT), and H4 (AC) were transfected into MLTC-1 cell lines to assess their effect on the transcriptional activity of the *PLCz* promoter. The reporter plasmid with pGL3-H2 had higher relative luciferase activity than those with pGL3-H4 and pGL3-H1 (*P*<0.001). Bulls with the H2H2 haplotype combination had notably higher ejaculate volume, initial sperm motility, sperm density, and frozen semen motility than the H4H4 and H1H1 haplotype combinations, as well as lower sperm deformity rates than H4H4 and H1H1. A possible explanation for these results is the localization of *PLCz* expression. *PLCz* expression was found to be localized in the post-acrosomal region of murine, bovine, and human sperm [46]. The localization of *PLCz* in the post-acrosomal region at the head of the sperm is important because the head is rapidly exposed to the ooplasm after gamete fusion; *PLCz* is reported to have an oocyte-penetrating effect in the sperm acrosome for successful fertilization [47]. Furthermore, *PLCz* mRNA was significantly expressed in the testis and epididymis of G-I boars as compared with G-II boars; G-I boars are noted for their relatively better sperm quality [48]. The association analysis of the four haplotypes confirmed the observed luciferase activity. The different haplotypes exhibited different promoter activities, which implied that genetic variants are likely to regulate *PLCz* expression and consequently influence the physiological function of PLCz on semen quality traits. For example, promoter region polymorphisms in the human MBL2 gene were found to control the baseline expression of MBL2 [39]. The haplotypes HY, LY, and LX were correlated with high, intermediate, and low MBL levels, respectively [39], [49]. Therefore, future studies should identify natural genetic structures that could be changed or utilize artificial selection in Chinese Holstein bulls under various environmental or economic conditions. Thus, the frequency of favorable alleles in the population would be expanded, thereby gradually fixing the dominant alleles for high-quality sperm characteristics in future generations of Chinese Holstein cattle breeds.

Our findings suggest that the functional genetic variants of *PLCz* and their respective haplotypes modulate the transcriptional activity of the *PLCz* promoter and may contribute to the semen quality traits of Chinese Holstein bulls. Moreover, promoter fragments with different haplotypes exhibited different promoter activities, which imply that these genetic variants are likely to regulate *PLCz* expression and consequently influence physiological functions of the gene.
